# Prognostic Value of Stress-Induced Hyperglycemia in High-Acuity Emergency Department Patients

**DOI:** 10.3390/jcm15041618

**Published:** 2026-02-19

**Authors:** Aikaterini Apostolopoulou, Christos Kofos, Marios G. Bantidos, Sofia-Chrysovalantou Zagalioti, Sofia Gkarmiri, Anna Drokou, Christina Kaltsidou, Nikolaos Koumianakis, Aikaterini Letsiou, Eleni Panayi, Grigorios Voulgaris, Paraskevi Katrana, Alexandra Arvanitaki, Vasileios Grosomanidis, Efstratios Karagiannidis, Barbara Fyntanidou

**Affiliations:** 1 Department of Emergency Medicine, Aristotle University of Thessaloniki, AHEPA University Hospital, 54636 Thessaloniki, Greecesofia_zag@yahoo.com (S.-C.Z.); nkoumian@auth.gr (N.K.); letsioua@auth.gr (A.L.);; 2 Second Department of Cardiology, Aristotle University of Thessaloniki, Hippokration General Hospital, 54642 Thessaloniki, Greecealexandra.arvanit@gmail.com (A.A.); 3 Centre of Orthopaedic and Regenerative Medicine (CORE), Center for Interdisciplinary Research and Innovation (CIRI)-Aristotle University of Thessaloniki (AUTH), Balkan Center, Buildings A & B, Thessaloniki, 10th km Thessaloniki-Thermi Rd, P.O. Box 8318, 57001 Thessaloniki, Greece; 4 Department of Anesthesiology and ICU, Faculty of Medicine, Aristotle University of Thessaloniki, 54124 Thessaloniki, Greece

**Keywords:** acute illness, emergency department, mortality, prognosis, risk stratification, stress-induced hyperglycemia

## Abstract

**Background/Objectives**: Stress-induced hyperglycemia (SIH) is frequently observed in critically ill patients and has been associated with adverse outcomes in individuals both with and without known diabetes mellitus (DM). However, evidence regarding its prognostic utility for in-hospital mortality in high-acuity emergency department (ED) populations remains limited. **Methods:** We conducted a retrospective observational cohort study of consecutive adult ED patients classified as Emergency Severity Index (ESI) triage level 1. SIH was defined a priori as an admission serum glucose > 140 mg/dL, a pragmatic cutoff widely applied in clinical practice despite ongoing debate regarding optimal pathophysiological thresholds. Associations with in-hospital mortality were assessed using logistic regression in the overall cohort and stratified by DM status. Additional analyses assessed the prognostic performance of admission glucose as a continuous variable. **Results**: Of 470 included patients, 435 had complete mortality data; 247 (56.8%) died during hospitalization. SIH was present in 258/435 (59.3%)and known DM in 114/435 (26.2%). SIH was associated with higher in-hospital mortality in univariate analysis (OR 2.90, 95% CI 1.91–4.43; *p* < 0.001) and remained independently associated after adjustment (adjusted OR 2.22, 95% CI 1.41–3.51; *p* < 0.001). The association between SIH and mortality persisted in both non-DM and DM subgroups, with no significant interaction by DM status. SIH alone showed modest discrimination for mortality (AUC 0.625, 95% CI 0.572–0.669), whereas continuous admission glucose performed better. Discrimination improved in the multivariable model (AUC 0.728, 95% CI 0.677–0.779). Restricted cubic spline analysis demonstrated a strong overall association between admission glucose and mortality without evidence of nonlinearity, indicating an approximately linear risk increase across the observed glucose range. **Conclusions**: Regarding severely ill ED patients, classified as ESI triage 1, SIH is an independent predictor of in-hospital mortality irrespective of DM status. Admission glucose may improve early risk stratification when incorporated into clinical models.

## 1. Introduction

Acute hyperglycemia commonly accompanies critical illness and has gained attention for its clinical implications and pathophysiological relevance in contemporary acute care research [1]. Evidence consistently indicates that blood glucose levels measured within the first 24–48 h following admission are frequently elevated [2]. This early hyperglycemic response is widely recognized as a manifestation of stress-induced metabolic dysregulation rather than an indication of pre-existing or newly diagnosed diabetes mellitus (DM), and is thus defined as stress-induced hyperglycemia (SIH) [3]. As a readily obtainable and simple measurement, it can provide clinically meaningful information to healthcare practitioners and serve as an early warning signal, prompting heightened vigilance and targeted management in selected patient populations [4,5,6,7].

The mechanisms underlying SIH are complex and multifactorial [8]. Insulin sensitivity is markedly impaired by pro-inflammatory mediators, including cytokines and hepatokines such as interleukin-6 (IL-6), interleukin-1 (IL-1), and tumor necrosis factor-α (TNF-α) [9,10]. In parallel, activation of the hypothalamic–pituitary–adrenal (HPA) axis leads to increased cortisol secretion in response to physiological stress. Cortisol, together with catecholamines, glucagon, and growth hormone, suppresses pancreatic insulin secretion and exacerbates peripheral insulin resistance [8,11]. Catecholamines and cytokines—most notably glucagon—promote increased hepatic gluconeogenesis, further contributing to hyperglycemia. Additionally, Forkhead box O (FOXO) transcription factors have been implicated in the pathophysiology of SIH through their regulation of hepatic glucose metabolism and insulin signaling [12]. Altered FOXO activity influences the expression of gluconeogenic and hepatocyte-related genes, contributing to insulin resistance and dysregulated glucose homeostasis during critical illness [13].

While most evidence linking SIH with adverse outcomes derives from critically ill or intensive care unit (ICU) populations [5,6,7], where continuous monitoring and protocolized care prevail, there is increasing recognition that admission glucose metrics also carry prognostic information in ED settings. Prior ICU-based studies have shown SIH and related indices such as stress hyperglycemia ratio are independently associated with mortality across critically ill patients, highlighting their value as severity markers. In contrast, despite the severity of illness encountered in the ED, evidence regarding SIH in ED populations remains limited. Although the ICU and ED represent distinct clinical environments, both involve acute physiological stress and high illness acuity. Within this context, evaluating SIH in critically ill ED patients allows assessment of its prognostic utility at an early stage of patient care, where timely risk stratification is essential. To date, there is a notable lack of non-disease-specific ED studies that define patient populations according to triage acuity and evaluate SIH using contemporary analytical approaches. This gap is particularly relevant given the need for early, diagnosis-independent risk stratification in the ED.

The effect of SIH warrants evaluation across diverse patient categories, as it may play a significant role in outcome prediction irrespective of the underlying disease. There are indications of a possible association between SIH and increased mortality in trauma patients, although substantial uncertainty remains due to limited statistical robustness [14]. Moreover, SIH may have prognostic value in patients with acute large-vessel occlusion ischemic stroke, particularly regarding the development of cerebral edema and hemorrhagic transformation—both severe indicators of brain injury [15]. In addition, a study including patients with acute pancreatitis demonstrated that SIH adversely affects multiple clinical outcomes, including length of hospital stay, intensive care unit (ICU) admission rates, and an increased risk of infected pancreatic necrosis [16]. Finally, a meta-analysis of patients with sepsis admitted to the ICU showed that SIH, defined by early admission blood glucose levels, is associated with increased short-term mortality in non-DM patients [17].

The aim of the present study is to assess the predictive utility of SIH for clinical outcome, examining primarily mortality, among critically ill patients admitted to the Emergency Department of a tertiary hospital in Northern Greece.

## 2. Materials and Methods

### 2.1. Study Population and Eligibility Criteria

This retrospective observational cohort study included consecutive adult patients presenting to the emergency department between 2023 and 2025 who were classified as Emergency Severity Index (ESI) 1 [18], indicating the highest level of acuity and the need for immediate medical evaluation and intervention. Eligibility required an initial serum glucose measurement obtained at ED presentation. SIH was defined a priori as admission serum glucose concentration > 140 mg/dL. The threshold of >140 mg/dL was selected based on guideline-based definitions of hyperglycemia in hospitalized and acutely ill patients, where this level is widely recognized as clinically relevant [19]. It is acknowledged that alternative definitions of stress-induced hyperglycemia have been used in prior studies, including higher cutoffs such as ≥180 mg/dL and ≥200 mg/dL, particularly in ICU-based cohorts or studies focusing on treatment thresholds and long-term metabolic outcomes [19,20]. DM status was determined by a previously documented diagnosis in the electronic medical record. Patients with missing in-hospital mortality outcome data were excluded from regression and discrimination analyses. For multivariable modeling, patients with missing covariate laboratory data were excluded using a complete-case approach without imputation. No additional exclusion criteria were applied. The study population exclusively included ESI level 1 patients, representing the highest-acuity emergency department population. This focus distinguishes our analysis from prior ED studies that included mixed-acuity cohorts and allows for a more precise evaluation of stress-induced hyperglycemia in critically ill patients.

### 2.2. Baseline Characteristics and Statistical Analysis

Demographic characteristics, comorbidities, laboratory measurements obtained at ED presentation, and emergency diagnoses were extracted from the electronic medical record and summarized for the overall cohort and stratified by SIH status. Continuous variables were reported as median with interquartile range (IQR) and compared using the Mann–Whitney U test. Categorical variables were summarized as counts and percentages and compared using the χ^2^ test or Fisher’s exact test, as appropriate. Baseline laboratory variables were analyzed using complete-case methods without imputation.

Univariate associations with in-hospital mortality were assessed using logistic regression in the total cohort and in subgroups defined by DM status. Multivariable logistic regression was used to evaluate the independent association between SIH and in-hospital mortality in the overall cohort, adjusting for clinically relevant covariates. Because standard logistic regression models within DM subgroups demonstrated evidence of quasi-separation and the DM subgroup was relatively small, Firth penalized logistic regression was used to obtain stable and unbiased estimates for multivariable analyses in both DM and non-DM patients. Effect modification by DM was evaluated using a penalized logistic regression model that included an interaction term between SIH and DM status, adjusting for the same covariates used in the primary multivariable analysis. Given the low proportion of missing data, analyses were conducted using complete cases, as this approach was unlikely to materially affect the results. Odds ratios (ORs) with 95% confidence intervals (CIs) were reported.

Model discrimination was assessed with receiver operating characteristic (ROC) curves and area under the curve (AUC) estimates with 95% CIs calculated by DeLong’s test. ROC analyses were performed for SIH as a single predictor in the total cohort and in DM and non-DM subgroups. Multivariable ROC curves were generated using predicted probabilities from the final models (standard logistic regression in the total cohort and Firth penalized regression in subgroups). Continuous admission glucose was additionally evaluated as a predictor of mortality, and within each cohort, the AUC for continuous glucose was compared with that of binary SIH using paired DeLong tests. Optimal glucose thresholds were identified using the Youden index with corresponding sensitivity and specificity.

Calibration of the final multivariable model in the total cohort was assessed using calibration-in-the-large and calibration slope from a logistic calibration model based on the logit of predicted probabilities, as well as a decile-based calibration plot comparing the mean predicted risk with observed mortality.

To further characterize the relationship between admission glucose and mortality beyond binary SIH definitions, restricted cubic spline analyses were performed. Four knots were placed at the 5th, 35th, 65th, and 95th percentiles of the admission glucose distribution (86, 129, 189, and 386.65 mg/dL).

An unadjusted spline model was first fitted to characterize the univariable glucose-mortality association. Subsequently, a multivariable spline model was fitted, adjusting for age, sex, heart failure, hypertension, white blood cell count, aspartate aminotransferase, cardiac arrest, and septic shock.

In both unadjusted and adjusted models, the overall association between glucose and mortality and the nonlinear component were evaluated using Wald χ^2^ tests. In the absence of statistically significant evidence for nonlinearity, admission glucose was subsequently modeled as a linear continuous predictor, scaled per 10 mg/dL increase, to provide an interpretable effect estimate.

Effect modification by DM status was assessed by including interaction terms between admission glucose and DM status in both linear and spline-based models. For spline analyses, interaction terms were used to evaluate whether the shape of the association between admission glucose and in-hospital mortality differed according to DM status. Due to the limited sample size in the DM subgroup, DM-specific spline curves were generated from the interaction model and are presented for descriptive visualization only. All analyses were performed in R Studio 4.4.2 (R Foundation for Statistical Computing, Vienna, Austria).

## 3. Results

**Figure 1:**
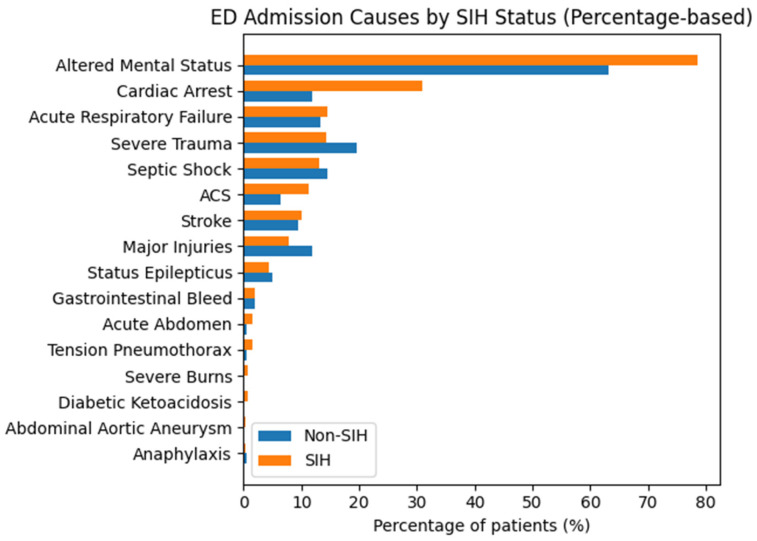
Admission causes by SIH status.

A total of 470 high-acuity ED patients were included. After excluding patients with missing outcome data, 435 patients comprised the outcome-complete cohort; 247 (56.8%) died in hospital, and 188 (43.2%) survived. SIH was present in 258 patients (59.3%), and known DM was present in 114 patients (26.2%) (Table 1). Compared to patients without stress-induced hyperglycemia, those with SIH were generally older and presented with greater metabolic and organ dysfunction, including lower pH values and higher creatinine and urea levels. Markers of physiological stress and tissue injury, such as white blood cell count, cardiac biomarkers, D-dimer, and liver enzymes, were also higher in the SIH group, and cardiac arrest and altered mental status were more frequent at presentation (Table 2, Figure 1). While diabetes mellitus was more prevalent among patients with SIH, most other chronic comorbidities and baseline hemodynamic parameters were broadly similar between groups.

In univariate analyses in the total cohort, SIH was strongly associated with in-hospital mortality (OR 2.90, 95% CI 1.91–4.43; *p* < 0.001) (Supplementary Table S1). In stratified univariate analyses, SIH remained significantly associated with mortality in both non-DM patients (OR 2.58, 95% CI 1.59–4.22; *p* < 0.001) and DM patients (OR 3.93, 95% CI 1.37–12.36; *p* = 0.013).

The primary multivariable model was fitted with adjustment for SIH, age, gender, heart failure, hypertension, white blood cell count, and SGOT. After adjustment, SIH remained independently associated with in-hospital mortality (adjusted OR 2.22, 95% CI 1.41–3.51; *p* < 0.001) (Table 3). Increasing age was independently associated with mortality. Hypertension demonstrated an inverse association with mortality in the adjusted model. No evidence of problematic multicollinearity was observed (all variance inflation factors < 1.3).

In stratified multivariable analyses using Firth penalized regression, SIH remained independently associated with in-hospital mortality in both non-DM patients (adjusted OR 1.96, 95% CI 1.15–3.36; *p* = 0.013) (Table 4) and DM patients (adjusted OR 3.45, 95% CI 1.25–10.41; *p* = 0.017) (Table 5). Among non-DM patients, increasing age and higher SGOT levels were also independently associated with mortality, whereas hypertension demonstrated an inverse association. Among DM patients, no additional covariates reached statistical significance.

Effect modification by DM status was assessed using a penalized logistic regression model including an interaction term between SIH and DM status. The interaction term was not statistically significant (adjusted OR 1.96, 95% CI 0.40–10.49; *p* = 0.41), indicating no evidence that the association between SIH and in-hospital mortality differed by DM status.

**Figure 2:**
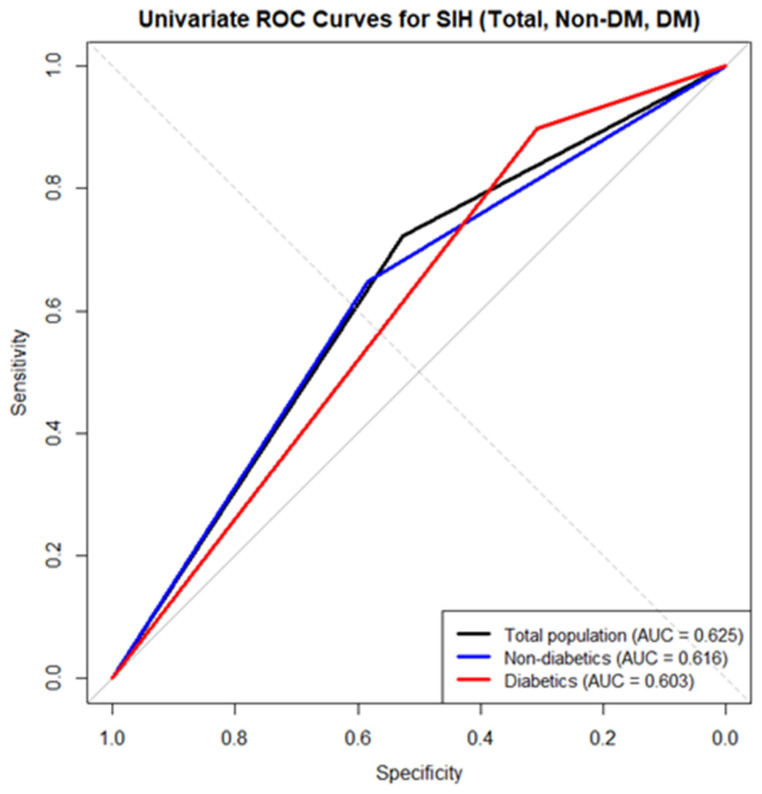
Receiver operating characteristic curves for stress-induced hyperglycemia as a univariate predictor of in-hospital mortality: black line—total cohort, blue line—non-DM patients, red line—DM patients.

**Figure 3:**
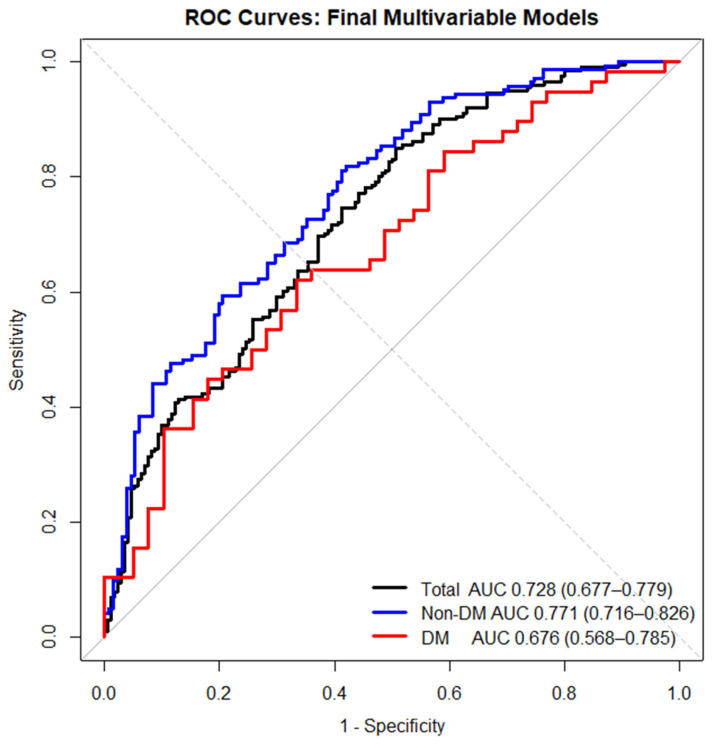
Receiver operating characteristic curves for multivariable models predicting in-hospital mortality: black line—total cohort, blue line—non-DM patients, red line—DM patients.

In ROC analyses, SIH alone demonstrated modest discrimination for in-hospital mortality in the total cohort (AUC 0.625, 95% CI 0.572–0.669), with similar performance in non-DM patients (AUC 0.616, 95% CI 0.558–0.673) and DM patients (AUC 0.603, 95% CI 0.520–0.686) (Figure 2). Within each cohort, continuous admission glucose demonstrated significantly greater discriminative ability than the binary SIH definition based on paired DeLong comparisons (total cohort: *p* = 0.0004; non-DM patients: *p* = 0.0166; DM patients: *p* = 0.0058). Exploratory analyses using the Youden index identified cohort-specific glucose thresholds associated with optimal discrimination, which differed between DM and non-DM patients (total cohort: 157.5 mg/dL; non-DM patients: 161 mg/dL; DM patients: 202.5 mg/dL). Discrimination improved when SIH was incorporated into multivariable models. Using predicted probabilities from the final models, the multivariable AUC was 0.728 (95% CI 0.677–0.779) in the total cohort, 0.771 (95% CI 0.716–0.826) in non-DM patients, and 0.676 (95% CI 0.568–0.785) in DM patients (Figure 3).

Calibration assessment of the final multivariable model in the total cohort demonstrated close agreement between predicted and observed risk. The calibration intercept was approximately 0, and the calibration slope was 1.00, indicating minimal overall miscalibration.

**Figure 4:**
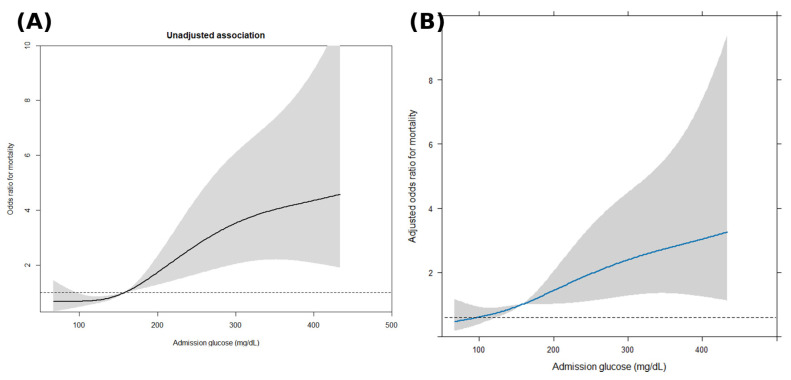
Restricted cubic spline analysis of the association between admission glucose and in-hospital mortality. (**A**) Unadjusted association. (**B**) Multivariable-adjusted association. The solid lines (black in panel A, blue in panel B) represent estimated odds ratios across admission glucose values. The gray shaded areas indicate 95% confidence intervals. The dashed horizontal line represents the reference value (odds ratio = 1).

In unadjusted RCS analysis, admission glucose demonstrated a strong monotonic association with in-hospital mortality (overall Wald χ^2^ = 32.15, df = 3, *p* < 0.0001), with no statistically significant nonlinear component (Wald χ^2^ = 4.00, df = 2, *p* = 0.14). As shown in Figure 4A, the odds of mortality increased progressively with higher admission glucose values across the observed range, with wider confidence intervals at extreme glucose levels.

After multivariable adjustment for age, sex, heart failure, hypertension, white blood cell count, aspartate aminotransferase, cardiac arrest, and septic shock, admission glucose remained independently associated with mortality (overall Wald χ^2^ = 17.18, df = 3, *p* = 0.0006), again without evidence of nonlinearity (Wald χ^2^ = 1.56, df = 2, *p* = 0.46), indicating an approximately linear relationship on the log-odds scale (Figure 4B). After adjustment, the strength of the association between admission glucose and mortality was reduced, as expected, but the overall pattern of progressively increasing risk with higher glucose levels remained unchanged. Clinically, this suggests that higher admission glucose levels are associated with a progressively increasing mortality risk, without an apparent glucose threshold beyond which risk changes abruptly.

Consistent with these spline findings, in the adjusted linear model, each 10 mg/dL increase in admission glucose was associated with higher odds of in-hospital mortality (adjusted OR 1.06, 95% CI 1.03–1.10; *p* < 0.001).

DM status did not modify the association between admission glucose and in-hospital mortality (interaction *p* = 0.90 in linear models; *p* = 1.00 in spline-based interaction tests). RCS curves derived from the interaction model demonstrated a similar, approximately linear increase in mortality risk with higher admission glucose among patients with and without diabetes. Although baseline mortality risk differed between groups, the shape and direction of the glucose-mortality association were comparable (Supplementary Figure S1). Confidence intervals widened at higher glucose values, particularly among patients with DM, reflecting a smaller subgroup sample size.

## 4. Discussion

This study examined the predictive value of SIH in a large and heterogeneous cohort of critically ill ED patients presenting with severe illness or trauma. The presence of SIH emerged as an independent predictor of mortality, conferring an almost threefold increase in the odds of in-hospital fatal outcomes after adjustment for demographic and clinically relevant covariates (including age, comorbidities, and acute illness severity markers). Importantly, this association remained consistent across univariate analyses, multivariable models adjusting for clinically relevant covariates, stratified analyses by diabetes status, and formal interaction testing, supporting the robustness of the observed relationship. These findings support SIH as a clinically meaningful and easily obtainable risk indicator in the ED setting, with potential utility for early risk stratification beyond traditional predictors. Because admission glucose is routinely available at the time of emergency department triage, assessment of stress-induced hyperglycemia can be seamlessly embedded within triage workflows, particularly for high-acuity patients. This enables early, diagnosis-independent risk stratification in high-acuity ED patients.

Although much of the SIH literature is diagnosis-specific, certain ED-related cohorts in heterogeneous populations similarly demonstrate that admission glucose is prognostically informative [21,22,23,24]. In non-traumatic critically ill ED resuscitation-room patients, Bernhard et al. reported a clear risk gradient with markedly higher mortality at glucose extremes, with breakpoints at ≤100 and ≥272 mg/dL [21]. Similarly, in broader “admitted-through-ED” cohorts, admission hyperglycemia has predicted mortality and length of stay, with the mortality signal most pronounced among patients without known diabetes [24]. Beyond mixed cohorts, comparable associations have been described in several acute conditions commonly encountered in high-acuity ED care (diagnosis-specific cohorts), including acute coronary syndromes, acute heart failure, ischemic and hemorrhagic stroke, pulmonary embolism, acute pancreatitis, pneumonia, and acute respiratory infection [25,26,27,28,29,30].

Stratification by DM status further reinforced SIH as an independent prognostic factor in both DM and non-DM patients. Although the magnitude of association appeared numerically higher in DM, the absence of a statistically significant interaction indicates that DM status does not materially modify the SIH-mortality relationship, supporting its use as a universal risk marker in critically ill ED populations. The robust association in non-DM patients, in particular, likely reflects an acute pathophysiological response to critical illness rather than underlying glycemic status (the chronic metabolic dysfunction of DM) alone, capturing risk that is not fully explained by traditional demographic or biochemical predictors.

The ROC analyses indicate that SIH, when defined as a binary variable, provides only modest discrimination for in-hospital mortality across the overall cohort and within DM and non-DM subgroups. From a clinical perspective, these AUC values indicate modest discriminative performance when SIH is used in isolation, which is expected for a single physiological parameter. Rather than serving as a standalone prognostic tool, SIH may be most clinically useful when integrated with established severity markers and risk scores to enhance early risk stratification in the ED. In contrast, admission glucose, considered as a continuous measure, demonstrated consistently better prognostic performance across all patient groups. This suggests that the relationship between acute hyperglycemia and adverse outcomes is graded rather than dichotomous, with risk increasing progressively as glucose levels rise. The identification of distinct, cohort-specific glucose thresholds using the Youden index further underscores the heterogeneity of stress hyperglycemia across metabolic backgrounds. Higher optimal cut-off values in DM patients likely reflect chronic glycemic adaptation and reduced vulnerability to moderate glucose elevations, whereas lower thresholds in non-DM patients suggest that even modest hyperglycemia may represent a more pronounced stress response and therefore carry greater prognostic significance. These Youden index-derived cutoffs should be interpreted as exploratory and cohort-specific and should not be applied as clinical decision thresholds without external validation in independent populations.

Beyond discrimination analyses, RCS modeling was used to formally evaluate the functional form of the association between admission glucose and in-hospital mortality. The spline analyses demonstrated a strong overall association but did not identify statistically significant nonlinearity, indicating that the relationship between glucose and mortality is approximately linear across the observed range.

Importantly, the absence of nonlinearity does not diminish the clinical relevance of stress-induced hyperglycemia as a prognostic construct; rather, it indicates that SIH captures a graded physiological stress response, whereby higher admission glucose levels are associated with progressively increased risk. In this context, modeling admission glucose as a continuous variable provides complementary insight into the severity spectrum underlying SIH, while preserving the practical value of SIH as a simple and clinically usable marker in the ED setting.

Finally, increasing evidence suggests that relative stress hyperglycemia may be better captured by indices incorporating baseline glycemic status [e.g., the stress hyperglycemia ratio (SHR) and the glycemic gap (GG)], rather than by absolute glucose thresholds alone [30,31]. Recent systematic reviews/meta-analyses support these indices, but evidence largely comes from admitted/ICU cohorts with routine access to extensive laboratory testing [32]. However, in the ESI-1 resuscitation setting, hemoglobin A1c (HbA1c) is typically not available at the time of triage/resuscitation, making SHR/GG difficult to implement for immediate decision-making; therefore, our results emphasize the pragmatic value of admission glucose as an ED-feasible risk marker. Furthermore, potential confounders such as pharmacological treatments and their timing should not be overlooked. The absence of detailed medication data in the overall sample precluded further analyses; however, factors such as insulin dosage and timing of administration, as well as the use and dose of vasopressors, may have influenced patient outcomes and could not be fully accounted for in the present study. The heterogeneity of clinical conditions reflects the complexity of the study population and underscores the need for future research to more comprehensively evaluate these treatment-related factors.

### 4.1. Study Strengths

The present study evaluated SIH in an unselected, triage-defined ED cohort. This non-pathology-restricted (all-comer ESI 1) design supports the general applicability of SIH as a point-of-care severity marker. At the same time, focusing on easily available and readily measurable indices, such as blood glucose, is of particular importance in the ED, where timely decision-making is critical and complex diagnostic tools may not be immediately accessible. This is complimented by the study’s focus on real-world practice, using routinely collected clinical and laboratory data obtained at presentation, which enhances external validity and generalizability. Moreover, stratified analysis across clinically relevant subgroups allowed for a more nuanced interpretation of the prognostic role of SIH, reducing the risk of oversimplified conclusions in a heterogeneous critically ill population. Lastly, by aligning SIH assessment with established ED triage systems, the study facilitates integration of glycemic markers into existing clinical workflows without adding complexity or delaying care.

### 4.2. Study Limitations

Despite its strengths, this study has several limitations. First, its retrospective design is inherently subject to selection bias and unmeasured confounding. Second, the lack of HbA1c values could be limiting the ability to distinguish acute stress hyperglycemia from previously unrecognized chronic hyperglycemia in some patients. In this context, the lack of data on specific diabetes medications represents a system-level limitation. In many cases, documentation at first patient contact was incomplete because life-saving interventions were appropriately prioritized in critically ill patients, which limited the availability of clinically detailed medication data and precluded more granular analyses. Third, despite a sizable overall cohort, subgroup and interaction analyses, particularly among patients with DM, were constrained by sample size, which may have reduced power to detect effect modification. Additionally, the observed inverse association between a history of hypertension and in-hospital mortality should be interpreted cautiously, as it may reflect residual confounding, treatment-related effects, or survivorship bias rather than a true protective effect. Finally, as a single-center study in a high-acuity ED population, generalizability may be limited, and external validation in independent cohorts is necessary before SIH can be adopted as a robust prognostic tool across broader ED populations.

### 4.3. Future Perspectives

Prospective, multicenter validation in comparable triage-defined high-acuity cohorts is warranted, with evaluation of whether admission glucose improves existing ED risk tools when combined with established severity markers (e.g., lactate and acid–base status) and dedicated risk scores [e.g., the Modified Early Warning Score (MEWS), the National Early Warning Score 2 (NEWS2), organ dysfunction scores/Sequential Organ Failure Assessment (SOFA), quick SOFA (qSOFA)] [33,34]. In centers where feasible, point-of-care HbA1c could be explored to test whether relative hyperglycemia indices (SHR/GG) add incremental value without compromising resuscitation timelines. If found to consistently provide meaningful incremental discrimination, routine early HbA1c measurement in triage-1 patients could be considered. Future work should also examine whether incorporating nonlinear glucose effects and early glucose trajectories (rather than a single value) improves discrimination and calibration in critically ill ED populations. Finally, while these findings support glucose as an early prognostic marker, they should not be interpreted as evidence that aggressive glucose lowering necessarily improves outcomes in this setting. For example, the 2021 Surviving Sepsis Campaign recommends initiating insulin therapy at a glucose level of ≥180 mg/dL (10 mmol/L), with a typical subsequent target range of 144–180 mg/dL (8–10 mmol/L); recommendations may evolve as new evidence emerges [33].

Although the findings are clinically important and demonstrate a strong association between stress-induced hyperglycemia and fatal outcomes, the present study should be interpreted within the context of prognostic research. Studies based on prognostic modeling are distinct from interventional clinical trials, which remain the cornerstone for establishing causal relationships and informing treatment strategies. Accordingly, these results should be interpreted with appropriate caution and should not be directly translated into clinical practice until validated by prospective interventional studies.

## 5. Conclusions

Early identification of SIH at ED presentation may provide incremental prognostic information across critically ill (ESI triage level 1) patients, irrespective of pre-existing DM. The consistency of its association with mortality in stratified and interaction analyses supports SIH as a robust marker of acute physiological stress and illness severity. Future prospective, multicenter studies should validate these findings and evaluate whether glucose-informed risk models improve clinical decision-making without delaying resuscitation workflows.

## Figures and Tables

**Table 1:** Baseline demographic, clinical, and laboratory characteristics of patients with and without stress-induced hyperglycemia.

Variable	Non-SIH (Median [IQR] or *n* (%)	SIH (Median [IQR] or *n* (%)	*p*-Value
**Age (years)**	63.00 [42.00–80.00]	70.00 [59.25–84.00]	**0.0007**
Systolic Blood Pressure (mmHg)	122.50 [100.00–136.25]	127.50 [96.75–160.00]	0.1146
Diastolic Blood Pressure (mmHg)	72.50 [62.00–85.75]	73.00 [60.00–88.00]	0.9325
Heart Rate (bpm)	90.00 [77.00–103.00]	90.00 [80.00–120.00]	0.1473
**pH**	7.29 [7.19–7.38]	7.18 [6.99–7.30]	**0.0015**
**Creatinine (mg/dL)**	1.01 [0.77–1.41]	1.26 [0.99–1.62]	**<0.001**
**Urea (mg/dL)**	37.00 [28.00–55.25]	46.00 [33.00–66.50]	**0.0006**
**hs-TnT (ng/mL)**	34.56 [11.00–88.24]	57.06 [20.77–174.00]	**0.0015**
CRP (mg/L)	2.08 [0.42–9.36]	1.21 [0.29–7.51]	0.1963
**Hemoglobin (g/dL)**	11.80 [10.12–13.47]	12.30 [10.60–14.30]	**0.0200**
**WBC (/μL)**	13.09 [8.55–37.45]	18.88 [12.52–84.92]	**<0.001**
PLT (μL)	223.00 [167.25–283.00]	204.50 [162.25–268.25]	0.1769
INR	1.09 [0.99–1.21]	1.12 [1.00–1.27]	0.1299
K (mEq/L)	4.20 [3.80–4.60]	4.10 [3.77–4.80]	0.8128
Na (mEq/L)	139.00 [136.75–141.00]	139.00 [136.00–142.00]	0.9539
**CPK (U/L)**	124.00 [55.75–308.00]	162.00 [79.00–406.50]	**0.0305**
**D-Dimers (ng/mL)**	776.50 [343.00–2922.50]	2332.00 [524.00–7055.00]	**<0.001**
**SGOT (IU/L)**	34.00 [22.00–65.00]	50.00 [29.00–105.75]	**0.0007**
Gender (male)	95 (59.4%)	152 (58.9%)	1.0000
Smoking	13 (27.1%)	21 (29.2%)	0.9670
Heart failure	6 (6.8%)	15 (12.1%)	0.3010
CAD	14 (15.4%)	27 (21.3%)	0.3581
Prior Stroke	11 (11.8%)	19 (14.5%)	0.7037
Hypertension	40 (41.7%)	66 (49.3%)	0.3153
Dyslipidemia	22 (23.2%)	36 (27.5%)	0.5618
**Diabetes Mellitus**	20 (13.2%)	87 (35.1%)	**<0.001**
Chronic Kidney Disease	6 (6.4%)	14 (10.6%)	0.3875
Known AF	14 (14.9%)	23 (16.9%)	0.8204
Liver disease	3 (3.2%)	2 (1.5%)	0.6521

**Table 2:** Emergency Department admission causes.

Variable	Non-SIH (Median [IQR] or *n* (%)	SIH (Median [IQR] or *n* (%)	*p*-Value
Cardiac Arrest	19 (11.9%)	79 (31.0%)	**<0.001**
Severe Trauma	31 (19.5%)	36 (14.2%)	0.1968
Acute respiratory failure	21 (13.2%)	37 (14.6%)	0.8595
Gastrointestinal bleed	3 (1.9%)	5 (2.0%)	1.0000
Status Epilepticus	8 (5.0%)	11 (4.3%)	0.9288
Septic Shock	23 (14.5%)	33 (13.0%)	0.7931
Anaphylaxis	1 (0.6%)	1 (0.4%)	1.0000
Tension Pneumothorax	1 (0.6%)	4 (1.6%)	0.6533
Stroke	15 (9.5%)	25 (10.0%)	1.0000
Abdominal aortic aneurysm	0 (0.0%)	1 (0.4%)	1.0000
Major Injuries	19 (11.9%)	20 (7.9%)	0.2281
Diabetic Ketoacidosis	0 (0.0%)	2 (0.8%)	0.5260
Severe Burns	0 (0.0%)	2 (0.8%)	0.5256
Altered Mental Status	96 (63.2%)	197 (78.5%)	**<0.001**
Acute Abdomen	1 (0.6%)	4 (1.6%)	0.6529
ACS	10 (6.3%)	28 (11.2%)	0.1355

**Table 3:** Multivariable logistic regression analysis for in-hospital mortality.

Variable	Adjusted OR	95% CI	*p*-Value
Stress-induced hyperglycemia (SIH)	2.22	1.41–3.51	0.001
Age	1.04	1.02–1.05	<0.001
Male gender	0.75	0.47–1.19	0.224
Heart failure	1.15	0.44–3.23	0.776
Hypertension	0.50	0.29–0.84	0.010
WBC	1.00	1.00–1.01	0.797
SGOT	1.01	1.00–1.02	0.028

**Table 4:** Multivariable Firth penalized logistic regression analysis for in-hospital mortality in non-diabetic patients.

Variable	Adjusted OR	95% CI	*p*-Value
Stress-induced hyperglycemia (SIH)	1.96	1.15–3.36	**0.013**
Age	1.04	1.03–1.06	<0.001
Male gender	0.66	0.38–1.15	0.139
Heart failure	0.84	0.19–3.71	0.808
Hypertension	0.35	0.17–0.68	**0.002**
WBC	1.01	1.00–1.01	0.763
SGOT	1.02	1.00–1.04	**0.018**

**Table 5:** Multivariable Firth penalized logistic regression analysis for in-hospital mortality in patients with diabetes mellitus.

Variable	Adjusted OR	95% CI	*p*-Value
Stress-induced hyperglycemia (SIH)	3.45	1.25–10.41	**0.017**
Age	1.02	0.98–1.06	0.372
Male gender	1.08	0.41–2.82	0.879
Heart failure	1.50	0.40–6.47	0.554
Hypertension	1.17	0.49–2.84	0.719
WBC	1.00	1.00–1.00	0.279
SGOT	1.01	1.00–1.01	0.671

## Data Availability

Study data will be available upon reasonable request from the corresponding study author (Efstratios Karagiannidis).

## Supplementary Materials

The following supporting information can be downloaded at: https://www.mdpi.com/article/10.3390/jcm15041618/s1. Table S1: Univariate associations with in-hospital mortality in the total cohort. Supplementary Figure S1: Restricted cubic spline analysis of the association between admission glucose and in-hospital mortality: (A) non-DM patients, (B) DM patients.

## Abbreviations

The following abbreviations are used in this manuscript: ACS: acute coronary syndrome; AF: Atrial fibrillation; AUC: area under the curve; CAD: coronary artery disease; CI: confidence interval; CKD: chronic kidney disease; CPK: creatine phosphokinase (creatine kinase); CRP: C-reactive protein; DBP: diastolic blood pressure; DKA: diabetic ketoacidosis; DM: diabetes mellitus; ED: emergency department; ESI: Emergency Severity Index; FOXO: Forkhead box O; GG: glycemic gap; HbA1c: hemoglobin A1c; Hgb: hemoglobin; HPA: hypothalamic–pituitary–adrenal; HR: heart rate; hs-TnT: high-sensitivity troponin T; ICU: intensive care unit; IL-1: interleukin-1; IL-6: interleukin-6; INR: international normalized ratio; IQR: interquartile range; K: potassium; MEWS: Modified Early Warning Score; Na: sodium; NEWS2: National Early Warning Score 2; OR: odds ratio; PLT: platelet count; qSOFA: quick Sequential Organ Failure Assessment; ROC: receiver operating characteristic; SBP: systolic blood pressure; SGOT: serum glutamic-oxaloacetic transaminase (AST); SHR: stress hyperglycemia ratio; SIH: stress-induced hyperglycemia; SOFA: Sequential Organ Failure Assessment; TNF-α: tumor necrosis factor-α; WBC: white blood cell count.
