# Supplementary material for: Prognostic Value of Stress-Induced Hyperglycemia in High-Acuity Emergency Department Patients
Source: J Clin Med. 2026 Feb 19;15(4):1618. doi: 10.3390/jcm15041618 (PMC12941850; doi:10.3390/jcm15041618)

Supplementary Content

Table S1. Univariate associations with in-hospital mortality in the total cohort.

**Predictor**	**Odds Ratio (OR)**	**95% CI**	**p-value**
Stress-induced hyperglycemia (SIH)	**2.90**	1.91 – 4.43	<0.001
Age	**1.03**	1.02 – 1.04	<0.001
Gender	**0.67**	0.45 – 0.98	0.042
Heart failure	1.58	0.63 – 4.20	0.339
Hypertension	1.26	0.75 – 2.13	0.381
Coronary artery disease	1.02	0.53 – 1.99	0.945
Chronic kidney disease	1.21	0.50 – 2.99	0.670
White blood cell count	1.00	1.00 – 1.00	0.431
SGOT (AST)	**1.01**	1.00 – 1.02	0.049

Supplementary Figure S1: Restricted cubic spline analysis of the association between admission glucose and in-hospital mortality: (A) non-DM patients, (B) DM patients.

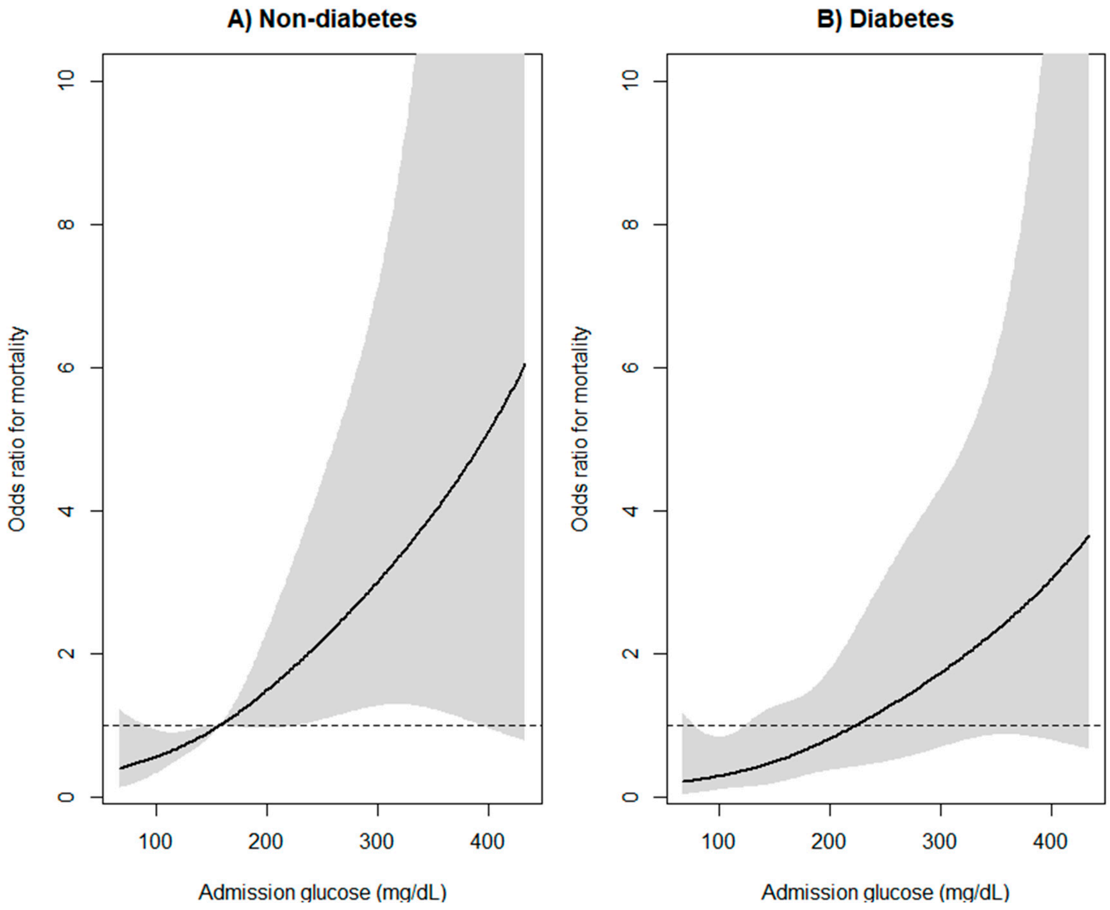

Supplement: Supplementary file 1 [file jcm-15-01618-s001.zip › jcm-4123846-supplementary.pdf]
